# On Being Ageist, Age-Friendly, Age-Inclusive: Differentiating Core Constructs and Their Implications

**DOI:** 10.1093/geroni/igab046.043

**Published:** 2021-12-17

**Authors:** Joann Montepare

**Affiliations:** Lasell University, Newton, Massachusetts, United States

## Abstract

The pioneering Age-Friendly University (AFU) initiative has called for institutions of higher education to respond to the needs of older, more age-diverse populations through new approaches to programs, practices, and partnerships. In exploring in more detail what it means for a campus to be age-friendly, the national AFU Inventory and Campus Climate Survey (ICCS) study has raised questions about how core theoretical concepts are defined and manifested. Using observations from the ICCS study, this presentation will discuss tensions among constructs (e.g., does being age-friendly indicate the absence of ageist attitudes; are age-inclusive practices by design age-friendly?) and how differentiating these constructs better can help higher education focus its efforts in more intentional and productive ways.

